# *Ab initio* simulation of diffractometer instrumental function for high-resolution X-ray diffraction^1^

**DOI:** 10.1107/S1600576715006986

**Published:** 2015-05-09

**Authors:** Alexander Mikhalychev, Andrei Benediktovitch, Tatjana Ulyanenkova, Alex Ulyanenkov

**Affiliations:** a Department of Theoretical Physics, Belarusian State University, Minsk, Belarus; b B. I. Stepanov Institute of Physics, National Academy of Sciences of Belarus, Minsk, Belarus; c Rigaku Europe SE, Ettlingen, Germany; d Atomicus GmbH, Karlsruhe, Germany

**Keywords:** diffractometer instrumental function, high-resolution X-ray diffraction, *ab initio* simulation

## Abstract

Modeling of the X-ray diffractometer instrumental function for a given optics configuration is important both for planning experiments and for the analysis of measured data. A fast and universal method for instrumental function simulation, suitable for fully automated computer realization and describing both coplanar and noncoplanar measurement geometries for any combination of X-ray optical elements, is proposed. The method can be identified as semi-analytical backward ray tracing and is based on the calculation of a detected signal as an integral of X-ray intensities for all the rays reaching the detector. The high speed of calculation is provided by the expressions for analytical integration over the spatial coordinates that describe the detection point. Consideration of the three-dimensional propagation of rays without restriction to the diffraction plane provides the applicability of the method for noncoplanar geometry and the accuracy for characterization of the signal from a two-dimensional detector. The correctness of the simulation algorithm is checked in the following two ways: by verifying the consistency of the calculated data with the patterns expected for certain simple limiting cases and by comparing measured reciprocal-space maps with the corresponding maps simulated by the proposed method for the same diffractometer configurations. Both kinds of tests demonstrate the agreement of the simulated instrumental function shape with the measured data.

## Abstract 2

A method for the simulation of the diffractometer instrumental function for high-resolution X-ray diffraction, applicable for coplanar and noncoplanar measurement geometry and for any combination of X-ray optical elements, is proposed. Good agreement is demonstrated between the measured and the simulated reciprocal-space maps, which account for the instrumental function.

## 1. Introduction   

Modeling of the X-ray diffractometer instrumental function for a given optics configuration is crucial both for planning experiments and for analyzing measured data. For practical applications, it is desirable to have a fast method of instrumental function simulation, suitable for fully automated computer realization and for the description of coplanar and noncoplanar measurement geometry for any combination of X-ray optical elements in a single universal way.

There are a number of approaches to the problem of instrumental function simulation. The fundamental parameters approach (Cheary *et al.*, 2004 ▶; Zuev, 2006 ▶; Masson *et al.*, 2003 ▶), based on analytical profile shape generation from physically based models, depending on principal parameters, has advantages such as adaptivity to any laboratory diffractometer and high speed of calculation. However, explicit expressions provided by this approach are only valid under a number of assumptions about measurement geometry (usually coplanar ones) and diffractometer configuration, and require a manual adaptation for new systems. Ray tracing (Lambert & Guillet, 2008 ▶; Rebuffi & Scardi, 2014 ▶), consisting of numerical consecutive tracing of rays from one optical element to another, represents a universal approach, well suited for computer implementation. Its main drawback is computational expensiveness, especially pronounced when a three-dimensional problem is considered (restriction of rays to the diffraction plane is not imposed). Another approach is empirical modeling of the diffractometer instrumental function by a simple fixed analytical function (Brügemann *et al.*, 1992 ▶; Sluis, 1994 ▶) or as a convolution of several simple (usually Gaussian) analytical functions (Gozzo *et al.*, 2006 ▶; Sabine, 1987 ▶) with optimizable parameters. This option provides simple analytical expressions and high speed of calculation at a cost of limited accuracy and applicability of simple models, and the need for model calibration using a reference sample.

Most of the research on instrumental functions is devoted to powder X-ray diffraction [see, for example, Masson *et al.* (2003 ▶), Cheary *et al.* (2004 ▶), Zuev (2006 ▶), Gozzo *et al.* (2006 ▶), Lambert & Guillet (2008 ▶), Rebuffi & Scardi (2014 ▶)]. This paper is focused on the less intensely discussed case (Brügemann *et al.*, 1992 ▶; Sluis, 1994 ▶; Boulle *et al.*, 2002 ▶; Kaganer *et al.*, 2001 ▶) of instrumental function modeling for high-resolution X-ray diffraction (HRXRD). The main aim is to design an instrumental function calculation procedure, combining advantages of both ray tracing and analytical approaches: universality and high speed of calculation. These features are encapsulated in a calculation algorithm that can be characterized as semi-analytical backward ray tracing. The detected signal is calculated as an integral of X-ray intensities for all of the rays reaching the detector. The integration is carried out over all spatial points of the detector and all the possible propagation directions of the rays. Taking into account not only angular but also spatial coordinates enables one to include the effects of finite source, sample and detector sizes and significantly improves the accuracy of the signal description for a two-dimensional detector. In order to provide method applicability for noncoplanar measurement geometry, the propagation of the rays in three-dimensional space is considered (without restricting the positions of the rays to the diffraction plane). To provide a high speed of calculation, the integration over two spatial coordinates describing the detection point is performed analytically, thus leaving for numerical treatment only two-dimensional integration over the ray directions. These principles provide a fast method for instrumental function calculation, applicable for noncoplanar as well as coplanar geometry, and for any combination of X-ray optical elements.

The paper is organized as follows. First, the main ideas underlying the proposed algorithm of instrumental function simulation are formulated. Then, a more detailed mathematical treatment of the problem is presented and the expressions for instrumental function calculation are derived. §4 provides a comparison of simulated reciprocal-space maps with their expected appearance for several simple limiting cases (open detector, highly divergent incident beam, nonmonochromatic beam). In §5, a comparison of the simulated and the measured reciprocal-space maps is presented.

## 2. Main principles   

### 2.1. Parameterization   

**Figure 1:**
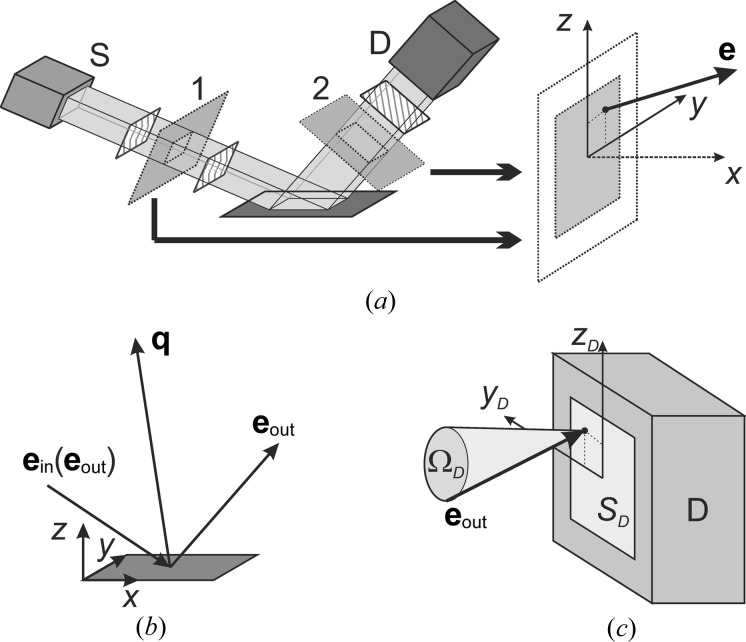
(*a*) Parametrization of radiation: for each cross section of the beam (planes 1 and 2 shown as examples), the axis *x* is parallel to the beam propagation direction; the axes *y* and *z* are perpendicular to the axis *x* and belong to the considered cross section. Each ray is characterized by its direction **e** and the coordinates *y*, *z* of the point where the ray hits the cross section. S is the X-ray source, D is the detector. (*b*) Idealized sample model: fixed scattering vector **q** provides complete determination of incident ray direction 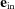 by the diffracted ray direction 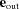. (*c*) Detected signal. 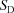 is the irradiated region of detector D; 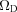 is the solid angle from which radiation reaches the detector.

In the considered model, the X-ray radiation in the diffractometer is characterized by the intensity distribution at each cross section of the beam. The coordinate system for beam cross sections is defined in the following way (Fig. 1 ▶ *a*). The axis *x* corresponds to the longitudinal direction of the beam (propagation direction of the central ray, which would be the only ray in the case of a nondivergent beam). The axes *y* and *z* correspond to the two transverse directions. For formulation of the proposed algorithm for simulation of the instrumental function their choice is irrelevant, but they must be fixed in a unique way for each cross section of the beam. In further consideration we fix these axes to represent the width (size in the diffraction plane) and height (or length, size perpendicular to the diffraction plane) of the beam, respectively, and to be parallel to corresponding dimensions of the source for the incident beam or the detector for the diffracted beam.

For such a choice of coordinate systems, the position of the cross section point is characterized by two variables, *y* and *z*. The propagation direction of a ray can be described by a normalized vector **e** = 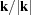 = 1, |**e**| = 1, where **k** is the wavevector associated with the ray. Because of the normalization, only two projections of the three-dimensional vector **e** are independent. In this paper, the projections **e**_*y*_ and **e**_*z*_ are used for ray characterization. Finally, the intensity per unit area of each cross section is represented as a function of two spatial and two angular coordinates, characterizing the considered spatial point (*y*, *z*) and propagation direction: 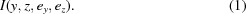



### 2.2. Optical elements   

For a vast number of X-ray optical elements, including all the elements commonly used for HRXRD, the beam intensity transformation has the form 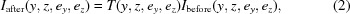
where 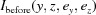 and 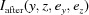 describe intensity distribution for cross sections closely before and after the considered optical element, and 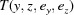 is the transmission function of the element.

Elements similar to a single-crystal (one-bounce) monochromator can also be taken into consideration by modifying the arguments of 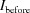 (in the same way as is done for the sample below). However, these elements are not used in the experimental setups considered in this paper. Therefore, for simplicity, we assume that equation (2) is valid for all the optical elements of the diffractometer. The parabolic multilayer mirror, used for collimation of the incident beam, does not fit this consideration in the form of a separate optical element and is described as a part of the X-ray source by modeling the source intensity distribution after the collimation (after the cross-beam optics unit) rather than immediately after the tube.

An important description property of optical elements commonly used in laboratory diffractometers for HRXRD (slits, Soller slits, monochromators) is their influence function on either the spatial or the angular part of the intensity distribution: for each element the transmission function depends either on *y* and *z* or on *e*_*y*_ and *e*_*z*_, but not simultaneously on all the four variables. This fact enables the classification of the elements as direction limiting (monochromator, Soller slits), for which 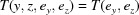, and coordinate limiting (slits) with 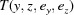 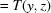. It is worth noting that this description is also suitable for a more general class of elements with a factorizable transmission function 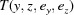 = 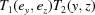: such elements can be represented as a combination of direction-limiting and coordinate-limiting elements with 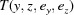 = 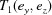 and 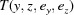 = 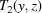, respectively.

The X-ray radiation source can be described in the same way. Formally, it can be considered as a dummy source of constant intensity 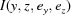 = 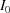 = constant, followed by an optical element with a transmission function corresponding to the real intensity distribution of the source: 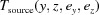 = 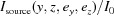. The introduction of a dummy constant intensity source is just a mathematical device for unification of the description of the elements, but it is not a real physical approximation: the equality 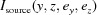 = 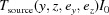 holds for any X-ray source according to the introduced definitions. Assuming the factorization of the source intensity as 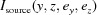 = 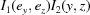, the optical element simulating intensity distribution can be represented as a pair of direction-limiting and coordinate-limiting elements.

To summarize, we make the following two assumptions about the optical elements used: (i) each element does not change ray directions and distances of all the rays from a certain central ray [equation (2)], and (ii) any element can be represented as a finite combination of direction-limiting and coordinate-limiting elements with a sufficient accuracy. The slits, apertures, Soller slits, two- and four-bounce channel-cut monochromators, X-ray sources with parabolic collimating mirrors, and cross-beam selection slits satisfy these conditions (or at least are equivalent to a set of optical elements satisfying these conditions). Single-crystal monochromators, dispersive double-crystal monochromators and beam compressors (Pietsch *et al.*, 2004 ▶) do not satisfy the first of the assumptions. These elements can be taken into consideration by adding a modification argument to equation (2). This modification influences the calculation speed insignificantly, but leads to a more complicated formulation of the simulation algorithm for the instrumental function and, for simplicity, is not included in this paper. Focusing capillary and bent-crystal optical elements (except for a Göbel mirror which is considered as a part of the X-ray source) do not satisfy the second condition. These elements are rarely used in HRXRD (they are used in powder diffraction and micro X-ray fluorescence analysis) and, therefore, we do not include them in the proposed simulation method here.

Within the discussed assumptions, equation (2) provides the unified description of all diffractometer elements (including source), except for the sample and the detector, which both need to be considered separately.

### 2.3. Idealized sample   

For calculation of the instrumental function, we consider an idealized model of a sample dealing with only a fixed scattering vector (Fig. 1 ▶ *b*) 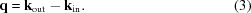
The diffraction pattern of a real sample can be reconstructed by convoluting the instrumental function, calculated for a fixed vector **q**, with the diffracted intensity depending on this vector.

For making the dependence between incident and diffracted wavevectors explicit, it is convenient to parameterize these vectors in the following way: 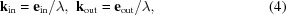
where λ is the wavelength associated with the considered ray (a nonmonochromatic beam may include rays with different values of λ), and 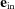 and 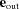 are unit vectors characterizing the direction of the ray before and after diffraction at the sample.

Equations (3) and (4) together with normalization conditions for unit vectors 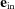 and 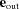 imply that for a fixed scattering vector **q** both the direction of the incident ray 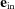 and the wavelength λ are determined in a unique way by the direction of the diffracted ray: 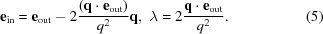



Therefore, in the considered model the wavelength and the propagation direction at any cross section for each ray are completely defined by specifying the propagation direction of the ray in the detector arm of the diffractometer.

### 2.4. Detected signal   

The detected signal is proportional to the radiation flux irradiating the detector, which in its turn is equal to the integral of the intensity distribution 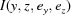 at the beam cross section at the detector window over the area of the detector and over all possible propagation directions (Fig. 1 ▶ *c*): 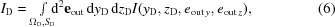
where Ω_D_ is the solid angle from which radiation penetrates the detector and *S*_D_ is the irradiated area of the detector. From a physical point of view, this expression can be interpreted as the sum of intensities of all the beams reaching the detector.

The transverse size of the detected beam is limited by the receiving optics rather than by the size of the detector itself. The detected beam divergence is also restricted by the receiving and (or) the incident optics and is rather a small value. Therefore, it is strongly inefficient to carry out the numerical integration in equation (6) over the whole area of the detector and the whole solid angle from which the radiation can reach the detector in principle. To reduce this inefficiency, the actual integration limits in further calculations are provided by a spatial and angular beam shape estimation for the considered diffractometer configuration. This estimation can be carried out by gathering inequalities, imposed by each optical element on the variables *y*, *z*, **e**_*y*_, **e**_*z*_ and, therefore, due to the relations (5), on the integration variables 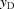, 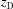, 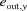, 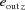.

## 3. Mathematical formulation of calculation method   

### 3.1. Description of optical elements   

According to the procedure outlined above for the simulation of instrumental function, each optical element of the diffractometer is characterized by its transmission function 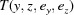 and a set of inequalities, imposed by the element on the variables *y*, *z*, *e*_*y*_, *e*_*z*_ (the inequalities provide the borders of the regions of the nonzero transmission function values). The models for the optical elements provided below describe the standard optics of a conventional commercial diffractometer.

The transmission function of slits is modeled by a rectangular distribution: 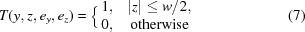
for a width-limiting slit of width *w* and 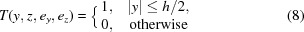
for a height-limiting slit of height *h* (here width corresponds to the *z* direction, lying in the diffraction plane, and height corresponds to the *y* direction, perpendicular to the diffraction plane). This form of transmission function implies the following inequalities for the beam-shape estimation after the slits: 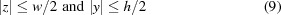
for width-limiting and height-limiting slits, respectively.

The Soller slits (including parallel slit analyzers and parallel slit monochromators) are described by transmission functions 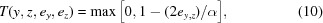
where the index *y* or *z* depends on the orientation of the parallel plates’ direction and α is the angular resolution of the Soller slits. The inequalities describing beam shape after these optical elements are 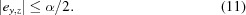



**Figure 2:**
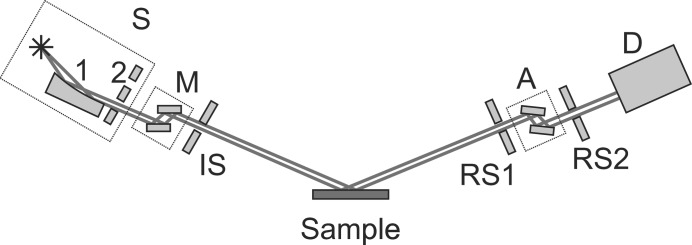
Scheme of the diffractometer configuration: S is the X-ray source [including parabolic multilayer mirror (1) and a cross-beam optics unit with selection slit (2)], M is the incident-beam crystal optics [a two-bounce monochromator in (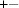) arrangement is shown], IS is the incident slit, RS1 and RS2 are the receiving slits, A is the diffracted-beam crystal optics [a two-bounce analyzer in (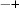) arrangement is shown], and D is the detector.

The transmission functions of the monochromators are modeled on the basis of the dynamical theory of diffraction (Authier, 2001 ▶) and for a two-bounce monochromator the transmission function is 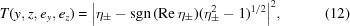
where 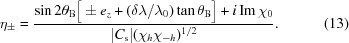


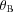
 is the Bragg angle of the used reflection **h**, 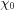, 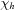 and 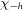 are Fourier components of polarizability, 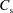 is the polarization factor (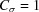 for σ polarization and 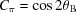 for π polarization), and λ_0_ is the average wavelength of the X-ray beam; 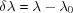 describes the difference between wavelength λ associated with the considered ray and described by equation (5) and the average wavelength λ_0_. The 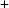 sign denotes (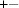) arrangement of the monochromator (Fig. 2 ▶), whereas the 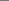 sign corresponds to (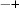) arrangement (usually used for the analyzer). The region of nonzero values for this kind of transmission function is described by the following inequality: 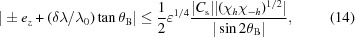
where 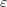 is a threshold for effectively zero values (values that are less than 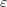 are treated as zero). For an accurate simulation of the laboratory diffractometer instrumental function the value 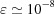 is usually sufficient.

The transmission function of a four-bounce monochromator is equal to 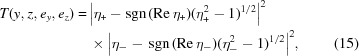
where the same notation is used as that given in equation (12). The beam shape after this element is determined by the following two inequalities: 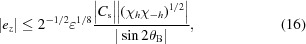


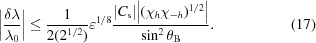



The dependence of the transmission functions of monochromators on the polarization of rays implies that the calculation of the instrumental function must be carried out separately for σ and π polarizations. The diffraction profiles from real samples depend on the polarization, too. Therefore, the instrumental functions corresponding to two polarizations must be convoluted with the diffraction profiles separately, and after the convolution the result can be averaged over the polarization of the incident beam.

For an X-ray source (including parabolic multilayer mirror, if used) the angular and spatial intensity distributions are assumed to be independent. The spatial part of the distribution is modeled by a rectangular shape (for example, corresponding to the transmission function of the selection slit of the cross-beam optics). The angular part of the distribution is taken in the form of a Lorentzian contour for parallel beam geometry (when a parabolic mirror is used) or a broad rectangular distribution, limited by the width and height of the selection slit of the cross-beam optics unit, in the configuration without a collimating parabolic mirror.

The finite size of the sample is taken into account by its transmission function (actually determining diffraction on the sample) 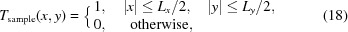
where 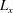 and 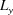 are the dimensions of the sample, and *x* and *y* are the coordinates of the point where the considered ray hits the surface of the sample in the coordinate system, as defined in Fig. 1 ▶(*b*). The ray is successfully diffracted by the sample when 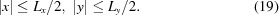
These inequalities describe the influence of the finite size of the sample on the shape of the beam.

### 3.2. Separation of integration variables for detected signal   

**Figure 3:**
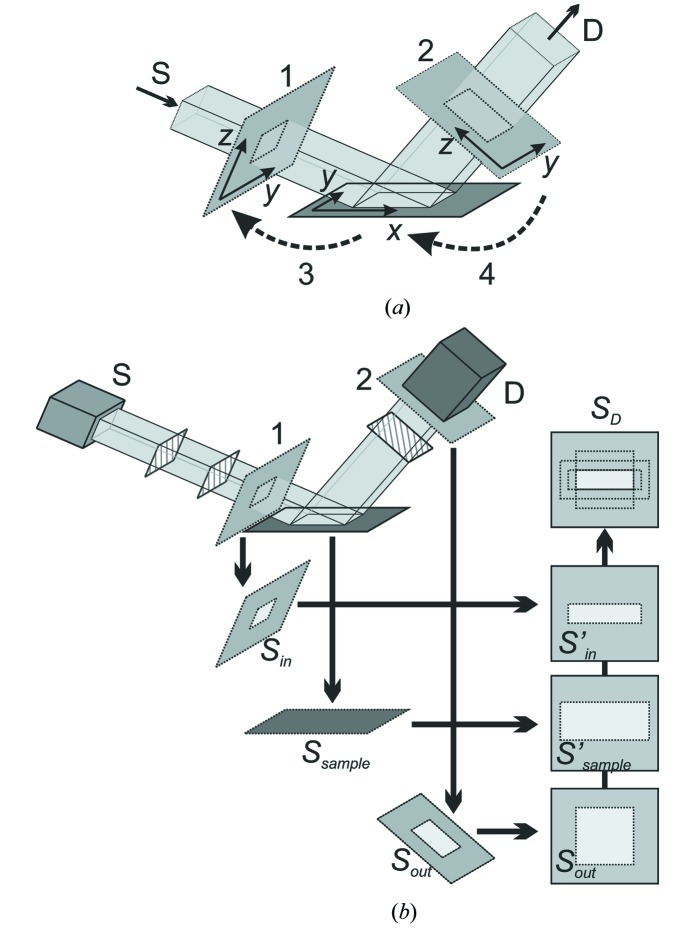
(*a*) The coordinate transformations between the sample coordinate system and the coordinate systems of the reference cross section (1) of the source arm (situated immediately before the sample) and the cross section (2) of the detector arm situated immediately after the sample. Transformations (3) and (4) are described by matrices 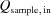 and 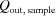, respectively. S is the X-ray source, D is the detector. (*b*) Scheme for calculation of the detector area irradiated by rays with fixed direction (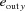, 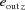). Regions of nonzero transmission function values of all coordinate-limiting elements of the source and the detector arms are projected onto reference cross sections (1) and (2), respectively. Then the regions of nonzero values of the transmission function for the source arm 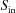 and the sample 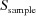 are projected onto the detector reference cross section. The irradiated region of the detector is formed by the intersection of the two projected regions and the region of nonzero transmission function values for the detector arm 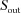.

In order to use the derived characteristics of the optical elements for the detected signal calculation, one can represent the integrand of equation (6) for the detected signal 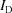 in the following form: 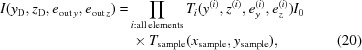
where for each optical element the spatial and angular coordinates are determined in a unique way by the considered ray (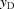, 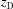, 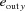, 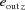): 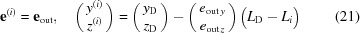
for the elements of the detector arm of the diffractometer, where 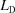 is the position of the detector (distance from the sample stage) and 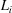 is the position of the *i*th optical element: 
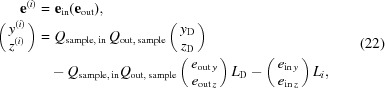
for the elements of the source arm, where the dependence of 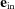 on 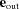 is provided by equation (5); matrices 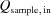 and 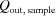 describe the transformation from the coordinates (*x*, *y*) of the sample surface to the coordinates (*y*, *z*) of the source arm cross section immediately before the sample and from the coordinates (*y*, *z*) of the detector arm cross section immediately after the sample to the sample coordinates (*x*, *y*) as shown in Fig. 3 ▶(*a*). The coordinates of the ray diffraction point at the sample, used for calculation of the sample transmission function, are determined by the following expression: 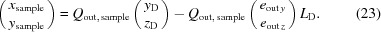



By separation of direction- and coordinate-limiting elements, equation (20) can be transformed in the following way: 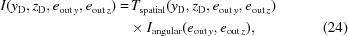
where 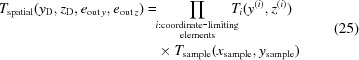
describes the total transmission function of the coordinate-limiting elements and 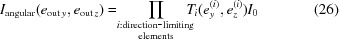
characterizes the intensity affected by direction-limiting elements only. Here, the independence of parameters 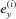 and 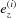 from 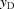 and 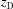, provided by equations (21)–(23), is taken into account.

Substitution of equation (24) into equation (6) provides the following expression for the detected signal: 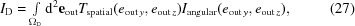
where, as it is shown below, integration in the spatial part of the transmission function 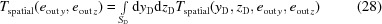
can be carried out analytically.

### 3.3. Analytical integration of spatial part of transmission function   

The transmission function of all coordinate-limiting elements mentioned above (slits, dummy optical element for modeling source intensity distribution, finite-size sample) are described by a rectangular distribution. The transmission of any of these elements is either 0 or 1 for each ray. The same property holds for the product of transmission functions of all the coordinate-limiting elements 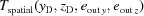. This means that the spatial part of the transmission function 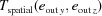 is equal to the detector area irradiated by the rays with fixed direction (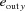, 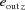). This area can be calculated as follows. For simplicity, we assume that the transmission function of the *i*th coordinate-limiting element has nonzero values in a rectangular region^2^

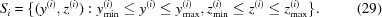
For pinholes and apertures, which have nonrectangular regions of nonzero transmission, only a slight geometrical modification of the expressions derived below is required. A certain ray direction (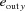, 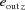) provides a singular one-to-one correspondence between spatial points of different beam cross sections. Therefore, all the regions from the source and the detector arms, treated separately, can be projected along rays onto certain fixed cross sections of the corresponding arm. For the source arm we choose the cross section immediately preceding the sample as the reference one (Fig. 3 ▶ *b*). For the detector arm, the reference cross section is chosen to correspond to the window of the detector.

The projection of the points (*y*, *z*) of a cross section of the source arm, situated at a distance *L* from the sample stage, onto the reference cross section along the chosen direction for the rays is described by the following transformation:^3^

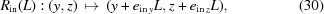
where the propagation direction 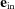 is determined by equation (5) in a unique way for fixed 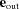. The analogous projection for the detector arm is defined as 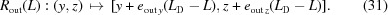
The shift transformations 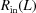 and 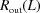 preserve the rectangular shape of regions 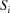. The spatial part of the transmission function for each of the two diffractometer arms, when recalculated to the reference cross sections, has nonzero values in rectangular regions^4^

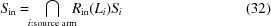
and 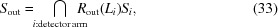
formed by the intersection of corresponding projections of the rectangular regions of the elements.

To calculate 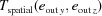, the region of nonzero values of the transmission function for the source arm 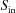, as well as the corresponding region for the sample 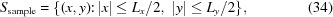
must be projected onto the detector reference cross section along the same rays. The results of this transformation are 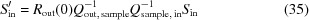
and 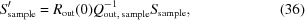
respectively. Finally, the region of the detector irradiated by rays with the fixed direction (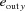, 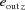) is formed by the following intersection of three regions: 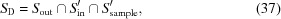
where 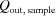 and 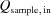 are the transformations discussed after equation (22). The spatial part of the transmission function is equal to the area of the region 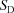: 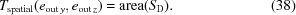
All of the described procedures required for the construction of region 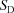 and the calculation of its area are elementary and provide significant speed-up in comparison with the straightforward numerical integration given in equation (28).

In practice, the calculation of 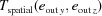 according to equation (38) takes about three times more operations than calculation of integrand 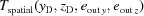 in equation (28). Assuming that at least 15 points per dimension are required for accurate enough numerical integration, we arrive at the speed-up of about 75 times in comparison with the common ray-tracing approach for simulation of individual rays, rather than simultaneous analysis of groups of rays with a selected propagation direction.

### 3.4. Beam shape estimation   

The constraints posed by each optical element on the beam shape are described by equations (9)–(19) and can be presented in the form of several linear inequalities for variables (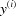, 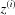, 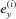, 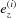), where *i* is the number of the considered element, and, owing to the linearity of relations, for integration variables (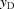, 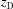, 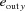, 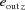) 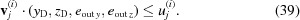



The angular integration limits in equations (6) and (27) can be formulated as 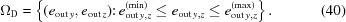
The strict limits 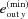, 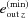, 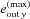, 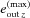 of the region, determined by the system of inequalities (39), can be found by the method of subdefinite calculations (Babichev *et al.*, 1993 ▶; Semenov *et al.*, 1997 ▶). The idea of the method is to transform the system of *N* inequalities 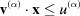 for *M* variables 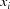 into a system of *MN* explicit constraints of the form: 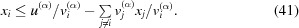
The iterative application of these constraints enables one to determine the maximally tight bounds **x** ^(min)^ and **x** ^(max)^ (Semenov *et al.*, 1997 ▶).

To estimate the simulation speed-up provided by the accurate beam shape estimation instead of integration over all possible directions of rays reaching the detector, we consider the modeled HRXRD setup with a four-bounce Ge(220) monochromator, a 1 mm width-limiting incident slit, a flat sample and a 20 × 20 mm detector at a distance of 300 mm from the sample. A rough estimate of the beam divergence (integration limits for angular variables) can be found as the detector width divided by the distance from the width-limiting slit along the beam (about 3°). The value found by accurate beam shape estimation is 0.02° (including tails of the distribution) in the considered case. Therefore, the described procedure is able to decrease the size of the integration region by a factor of 150 (or 20 for a setup with a two-bounce monochromator), which leads to a significantly smaller number of integration points with the same accuracy of the final result.

### 3.5. Algorithm of instrumental function simulation   

Summarizing the results obtained above, the following algorithm of instrumental function simulation for a given diffractometer configuration and an ideal sample, which accepts a fixed scattering vector **q**, can be formulated. For each goniometer position, corresponding to a single point on a scan or on a reciprocal-space map, the following sequence of steps has to be taken to calculate the detected signal:

(*a*) Beam shape estimation: inequalities of the form (39) are gathered from all the optical elements (including the sample); the angular integration region 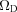 [equation (40)] is determined by the method of subdefinite calculations.

(*b*) Integration over the propagation directions of the rays: integration over angular variables 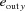 and 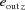 in equation (27) is carried out numerically with the integration limits determined by the region 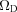. Estimations and practical trials for characteristic Lorentzian and rectangular shapes of the integrand function show that accurate results are obtained when at least 15–40 points are used for each of the integration dimensions. All of the next steps correspond to integrand evaluation and must be performed for each point (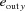, 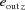).

(*c*) Calculation of the angular part of the intensity: for a fixed ray direction 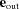, its directions 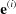 at the cross sections, corresponding to each direction-limiting element, are calculated on the basis of equations (21) and (22); the angular part of the intensity 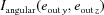 is then determined by equation (26).

(*d*) Calculation of the spatial part of the transmission function 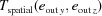: rectangular regions 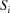 [equation (29)] of nonzero transmission function values are found for all of the coordinate-limiting elements and projected onto corresponding reference beam cross sections [equations (32) and (33)]. Transformation matrices 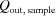 and 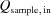 are calculated for the considered measurement geometry and goniometer positions (these matrices do not depend on 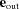 and can be calculated only once). The irradiated detector region 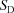 is determined by equation (37), where equation (34) is used for 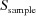. The spatial part of the transmission function 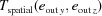 equals the area of the region 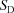. 

(*e*) The integrand value is formed by multiplication of 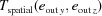 and 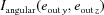.

In order to take into account the dependence of the transmission functions of the monochromators on the polarization of the radiation, the calculation procedures should be performed twice for two different polarizations, thus yielding two kinds of instrumental function 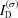 and 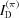, corresponding to σ and π polarizations and being simulated separately.

### 3.6. Convolution for a real sample   

The calculated value of detected signal 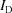 corresponds to an idealized sample, accepting fixed scattering vector **q**. For a real sample, integration over nonzero diffraction profile values, 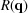, must be performed: 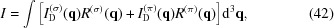
where the dependence of the diffracted signal intensity 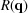 on the polarization is taken into account. For the case of epitaxial layers without diffuse scattering this expression is simplified: 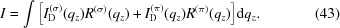



For effective numerical calculations, the actual integration limits for 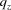 (or **q**) must correspond to the region of nonzero integrand values. These limits can be estimated by the method of subdefinite calculations for an extended system of inequalities. This extension by adding an external parameter μ transforms inequality 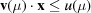 into^5^

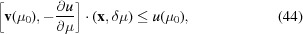
where 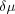 is a small variation of the parameter μ from its value μ_0_. The scattering vector **q** represents an external parameter for pure instrumental function simulation. Therefore, the region of nonzero values of 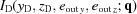 can be estimated by applying the method of subdefinite calculations to the system of inequalities, extended by adding variations of **q** around its initial value (for example, **h** for the considered reflection). The limits found after this procedure will correspond to the region of nonzero values of 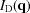 and, therefore, to the desired effective integration limits.

## 4. Physical consistency of simulation algorithm   

To verify the correctness of the proposed method for instrumental function simulation, we consider several simple limiting cases of diffractometer configurations. These configurations correspond to separated effects of finite detector resolution, incident beam divergence and radiation nonmono­chromaticity. Reciprocal-space maps (RSMs) of the pure instrumental function (corresponding to the above-described model of an ideal sample) are simulated for a coplanar out-of-plane measurement geometry (Fig. 2 ▶). The scattering vector **q** corresponds to the 224 reflection of a (001)-oriented perfect crystal of silicon.

**Figure 4:**
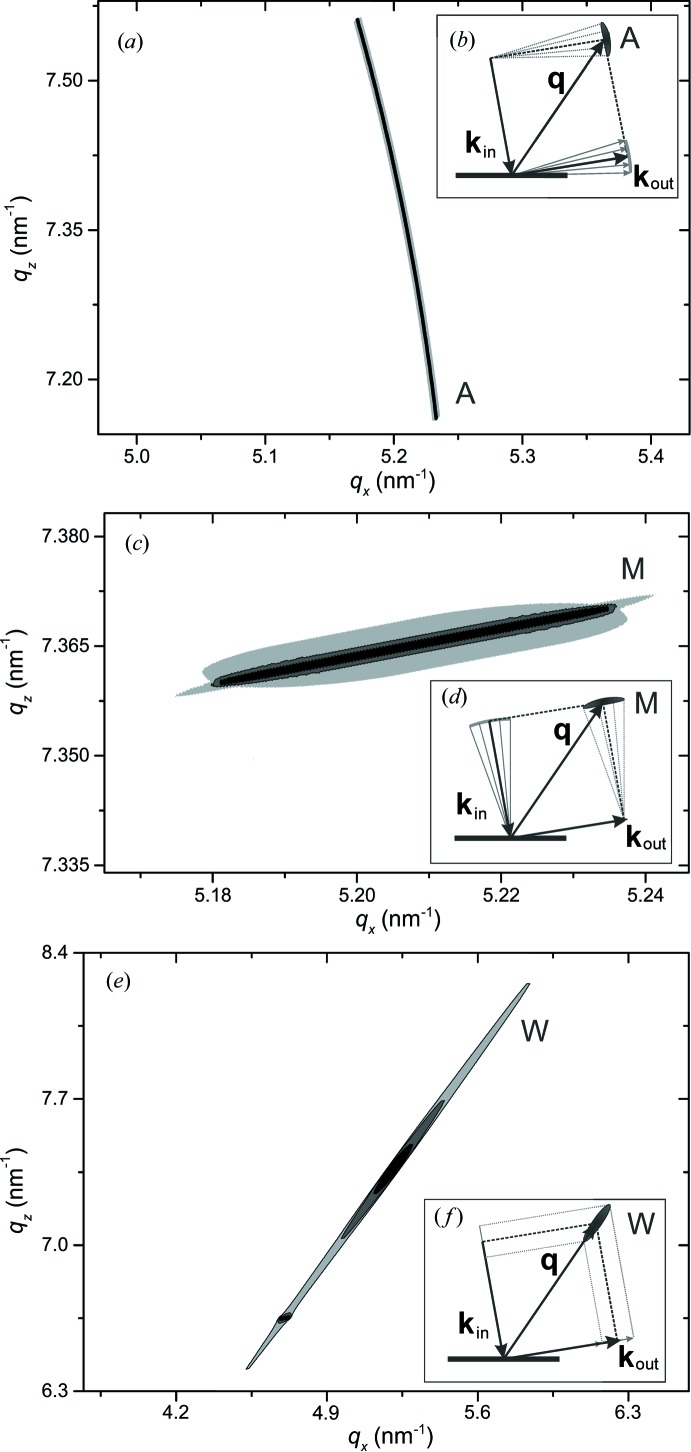
Simulated RSMs, showing the effects of finite angular resolution of receiving optics (*a*), incident beam divergence (*c*) and beam nonmonochromaticity (*e*). The ideal sample model with **q** corresponding to the 224 reflection of (001)-oriented silicon is used. Insets (*b*), (*d*) and (*f*) show geometrical schemes of streak formation. M is the monochromator streak, A is the analyzer streak and W is the wavelength streak. All RSMs presented in this paper use log-scale for intensity.

The effect of finite receiving optics angular resolution can be tested by using the following open-detector diffractometer configuration (see Fig. 2 ▶ for notation of optical elements). The monochromaticity and small angular divergence of the incident beam are provided by a four-bounce Ge(004) monochromator. The spatial size of the incident beam is constrained by a 0.05 mm width-limiting incident slit. This configuration of the diffractometer source arm fixes the direction and the length of the incident wavevector 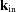 (Fig. 4 ▶ *b*). Therefore, the shape of the instrumental function map is almost completely determined by the inaccuracy of characterization of 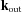 by the detector position. Fig. 4 ▶(*a*) shows that the simulated RSM has one streak, perpendicular to the direction of the diffracted wavevector 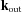, in complete agreement with the expected shape.

A similar effect of incident beam divergence can be simulated by using monochromatic radiation and fixing the direction of the diffracted wavevector 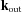. This result can be achieved by considering the configuration with a four-bounce Ge(004) analyzer and a 0.05 mm width-limiting receiving slit in the detector arm of the diffractometer. The simulated RSM (Fig. 4 ▶ *c*) exhibits one streak, perpendicular to the direction of the incident wavevector 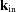, as expected (Fig. 4 ▶ *d*).

For modeling the effect of beam nonmonochromaticity with fixed directions of 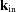 and 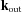, we consider the configuration without a monochromator or an analyzer. The small angular divergence of the incident beam and the high resolution of the detector arm are provided by using narrow incident and receiving slits (all the slits are 0.05 mm wide). The RSM simulated for this configuration of diffractometer is shown in Fig. 4 ▶(*e*) and exhibits one streak along **q** (Fig. 4 ▶ *f*).

**Figure 5:**
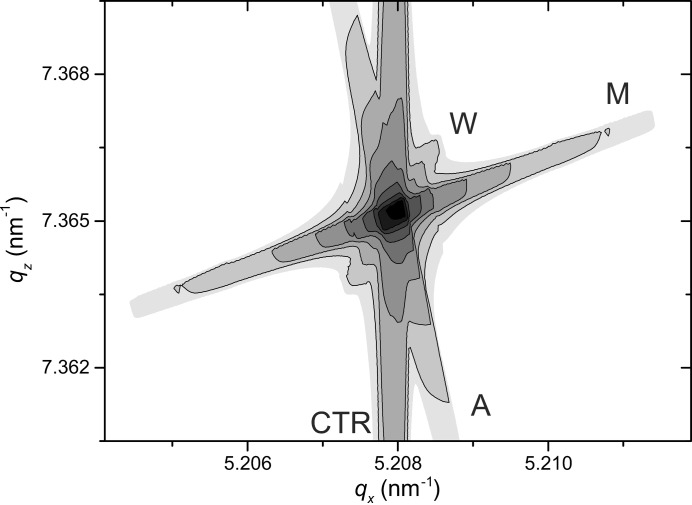
Simulated RSM with comparable effects of beam divergence, finite angular resolution of detector and beam nonmonochromaticity. The 224 reflection of ideal (001)-oriented silicon is considered. CTR is the crystal truncation rod, M is the monochromator streak, A is the analyzer streak and W is the wavelength streak.

Fig. 5 ▶ shows a simulated RSM for the configuration when all the above-discussed effects have similar orders of magnitude. The source arm contains a four-bounce Ge(004) monochromator and a 0.5 mm-wide incident slit. A two-bounce Ge(220) analyzer and a 1 mm-wide receiving slit are placed in the detector arm of the diffractometer. The RSM is simulated for the 224 reflection of a sample, modeled as a (001)-oriented perfect crystal of silicon, described by the dynamical theory of diffraction (Authier, 2001 ▶), and consists of four streaks: monochromator streak M, caused by incident beam divergence, analyzer streak A, corresponding to finite angular resolution of the detector arm, wavelength (radiation nonmonochromaticity) streak W and crystal truncation rod CTR of the sample.

## 5. Experimental testing of instrumental function simulation   

The second test of the proposed algorithm for instrumental function simulation consists of a comparison of simulated and measured RSMs with the same diffractometer configurations. For this purpose, several maps have been measured on a standard laboratory diffractometer for the 004 reflection of a (001)-oriented crystalline silicon sample.

**Figure 6:**
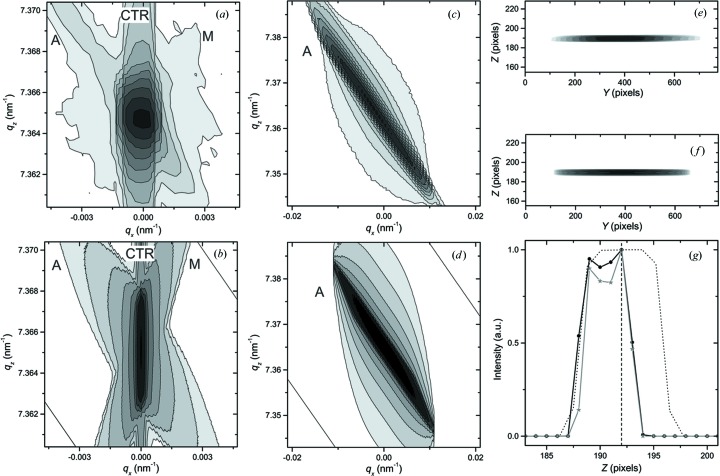
Measured (*a*), (*c*), (*e*) and simulated (*b*), (*d*), (*f*) maps [RSMs for (*a*)–(*d*), and diffracted beam intensity distribution maps for two-dimensional detector (*e*), (*f*)] for the 004 reflection of a (001)-oriented silicon sample. See text for explanations of the diffractometer configurations used. (*g*) Vertical distribution of the detected intensity for the maps (*e*), shown with a black solid line with circles, and (*f*), shown with a gray solid line with stars. Symbols (circles and stars) correspond to the positions and detected intensities of the detector pixels. The dotted line shows the intensity distribution for the same diffractometer configuration without source misalignment and the dashed line shows the central pixel of the detector.

Fig. 6 ▶(*a*) shows the RSM measured as 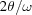
–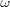 scans with a scintillation counter. A setup with a two-bounce Ge(220) monochromator and an analyzer, a 1 mm width-limiting incident slit, and 0.2 and 1 mm receiving slits is used (see Fig. 2 ▶ for a scheme of the diffractometer configuration). Fig. 6 ▶(*b*) shows the simulated map for this configuration with a sample modeled as a perfect crystal and described by the dynamical theory of diffraction. Both maps exhibit the analyzer (A) and the monochromator (M) streaks and crystal truncation rod (CTR), the reciprocal dimensions of which are in a good agreement.

The measured RSM in Fig. 6 ▶(*c*) and the simulated RSM in Fig. 6 ▶(*d*) were both measured as 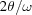
–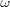 scans using a setup with a two-bounce Ge(220) monochromator, no analyzer, a 1 mm width-limiting incident slit, and 0.2 and 1 mm receiving slits. The maps are in a good agreement and both have a significant analyzer streak.

The maps in Figs. 6 ▶(*e*) and 6(*f*) correspond to coplanar measurement geometry with a two-dimensional detector. The source arm of the diffractometer contains a four-bounce Ge(220) monochromator and a 0.5 mm width-limiting incident slit. For easy comparison, the vertical distribution of detected intensity for the two maps is shown in Fig. 6 ▶(*g*). The asymmetry of the experimentally observed intensity distribution [solid black line in Fig. 6 ▶(*g*)] relative to the central pixel of the detector [dashed line in Fig. 6 ▶(*g*)] can be explained by a small misalignment of the used optics. A good agreement between measured and simulated profiles (both width and position of the peak) is achieved when a 0.2 mm downward shift of the source is assumed in simulation of the RSM in Fig. 6 ▶(*f*). The dotted line in Fig. 6 ▶(*g*) shows the simulated detected intensity distribution for the setup without the misalignment. The time required for simulation of RSMs in Figs. 6 ▶(*b*), 6(*d*) and 6(*f*) on a standard desktop computer is 40, 39 and 1 s, respectively.

The observed agreement between the measured and simulated maps proves the applicability of the designed simulation algorithm for the description of regular HRXRD measurements. The considered measurements correspond to a symmetric reflection in silicon samples. A more comprehensive testing of the algorithm for a wider group of different measurement geometries and scan types will be published elsewhere.

## 6. Conclusions   

To summarize, a novel algorithm for the simulation of the instrumental function is proposed and tested. According to the designed approach, X-ray radiation is represented as a set of rays, characterized by their propagation directions and the spatial points of their detection. Only the rays which arrived at the detector plate are considered. The detected signal is equal to the sum (integral) of intensities of all detected rays. A significant acceleration of the simulations is provided by performing an analytical integration over the spatial coordinates and by estimating the actual integration limits for angular variables by a subdefinite calculation method on the basis of inequalities describing the beam shape transformation by each optical element.

All procedures for simulation of the instrumental function are realized as algorithms and can be easily coded, which is crucial for their use in commercial software. For adding new optical elements, their transmission function and inequalities describing beam shape transformation have to be provided. Therefore, the set of described elements is not limited to the ones described in this paper, and can be easily extended. The designed method is universal and applicable for any diffractometer configuration and measurement geometry and provides a high-speed instrumental function simulation on a desktop computer. The proposed algorithm has been tested both for physical consistency and for agreement between simulated and measured reciprocal-space maps, and both approaches show the validity of the algorithm.

## Footnotes

2 Notation  stays for the set of all *values* for which the *condition* is satisfied.

3 Notation  defines *operator*, for which its action on any valid *value* is described by the correspondingly calculated *new value*.

4 Here, the symbol  describes the intersection of two-dimensional regions.

5 Notation  with -dimensional vector **a** and scalar *b* is used for -dimensional vector **c** with , , and .
